# Evidence of the Innate Antiviral and Neuroprotective Properties of Progranulin

**DOI:** 10.1371/journal.pone.0098184

**Published:** 2014-05-30

**Authors:** Hyeon-Sook Suh, Yungtai Lo, Namjong Choi, Scott Letendre, Sunhee C. Lee

**Affiliations:** 1 Department of Pathology, Albert Einstein College of Medicine, Bronx, New York, United States of America; 2 Department of Epidemiology and Population Health, Albert Einstein College of Medicine, Bronx, New York, United States of America; 3 Department of Neurology, University of California San Diego, San Diego, California, United States of America; University of Nebraska Medical Center, United States of America

## Abstract

**Background:**

Compelling data exist that show that normal levels of progranulin (PGRN) are required for successful CNS aging. PGRN production is also modulated by inflammation and infection, but no data are available on the production and role of PGRN during CNS HIV infection.

**Methods:**

To determine the relationships between PGRN and HIV disease, neurocognition, and inflammation, we analyzed 107 matched CSF and plasma samples from CHARTER, a well-characterized HIV cohort. Levels of PGRN were determined by ELISA and compared to levels of several inflammatory mediators (IFNγ, IL-6, IL-10, IP-10, MCP-1, TNFα, IL-1β, IL-4 and IL-13), as well as clinical, virologic and demographic parameters. The relationship between HIV infection and PGRN was also examined in HIV-infected primary human microglial cultures.

**Results:**

In plasma, PGRN levels correlated with the viral load (VL, p<0.001). In the CSF of subjects with undetectable VL, lower PGRN was associated with neurocognitive impairment (p = 0.046). CSF PGRN correlated with CSF IP-10, TNFα and IL-10, and plasma PGRN correlated with plasma IP-10. *In vitro*, microglial HIV infection increased PGRN production and PGRN knockdown increased HIV replication, demonstrating that PGRN is an innate antiviral protein.

**Conclusions:**

We propose that PGRN plays dual roles in people living with HIV disease. With active HIV replication, PGRN is induced in infected macrophages and microglia and functions as an antiviral protein. In individuals without active viral replication, decreased PGRN production contributes to neurocognitive dysfunction, probably through a diminution of its neurotrophic functions. Our results have implications for the pathogenesis, biomarker studies and therapy for HIV diseases including HIV-associated neurocognitive dysfunction (HAND).

## Introduction

Despite effective and widely available combination antiretroviral therapy (ART), HIV-associated neurocognitive disorder (HAND) affects up to 50% of HIV infected individuals [1]–[3]. HAND ranges in severity from very mild (asymptomatic neurocognitive impairment) to disabling (HIV-associated dementia (HAD)). HAD is associated with active HIV replication in the central nervous system (CNS) and neuropathological findings consistent with HIV encephalitis (HIVE). HIVE diagnosis requires the presence of multinucleated giant cells and microglial nodules signifying productive HIV infection. In the combination ART era, the prevalence of HAD/HIVE has decreased but the prevalence of the milder forms of HAND has increased. There is no clear understanding of the relationship between the virus and milder clinical manifestations of HAND. The levels of virus in brain poorly correlate with the degree of neurological impairment [4]–[6]. Moreover, cognitive deterioration can occur despite suppression of viral replication and immune recovery induced by ART. These changes could result from inadequate distribution of ART into the CNS with resulting residual replication in the brain, chronic CNS inflammatory and oxidative stress responses, or neurotoxic effects of treatment [1], [7]–[9]. Recent gene array studies also indicate that the pathophysiology of HAND may differ depending on the presence or absence of HIVE [10], [11], suggesting that clinical subtypes of HAND may exist based on detectable or undetectable HIV in the CSF. We have termed these conditions type I (detectable HIV in CSF) and type II (undetectable HIV in CSF) HAND based on the assumption that the mechanisms of CNS injury fundamentally differ in these two divergent environments. Several studies have focused on identifying biomarkers to monitor HIV immunopathogenesis in patients on ART, but none has yet proved reliable for diagnostic or therapeutic purposes [12]–[14]. While the role of neurotoxic viral and host factors released from HIV-infected macrophages has been investigated, detailed understanding of key molecules and cellular pathways that modulate HIV-macrophage (or microglia) interactions is lacking. Thus, there is an imperative need to understand the pathogenesis of HAND, especially the milder forms, and to identify new biomarkers for HAND.

Progranulin (PGRN) is a highly unusual molecule that is expressed by both neurons and microglia and has two seemingly unrelated functions: it is both a neuronal growth factor and a modulator of neuroinflammation [15]–[20]. PGRN gained much attention with the discovery of haploinsufficiency resulting from PGRN gene mutations leading to frontotemporal lobar degeneration, a fatal presenile dementing illness [21]–[23]. Several additional gene mutations (single nucleotide polymorphisms, SNP) that result in significant PGRN deficiency such as rs5848 have also been identified [24]–[26]. While neurons constitutively express PGRN, microglial cells are the primary source of regulatable PGRN in the brain [27]–[29]. Analysis of PGRN expression in systemic organs also demonstrated that tissue macrophages are the main expressors of PGRN with certain epithelial cells also expressing PGRN but at much lower levels [30]–[33]. PGRN expression in the two cell types is also differently regulated. For example, proinflammatory mediators such as TLR ligands and Th1 cytokines (IL-1/IFNγ) suppress PGRN production in (human) microglia but they upregulate PGRN in non-myeloid cells such as astrocytes and fibroblasts [34]–[36]. These results together support that PGRN is a neuronal growth factor and an immune modulator with cell-type-dependent regulation and function. The expression and function of PGRN are also species-dependent, as evident by the very subtle CNS pathology in homozygous knockout mice, in stark contrast to the fatal phenotype associated with haploinsufficiency in humans [37]. Furthermore, while in mice and mouse macrophages PGRN appears to function as a down modulator of cytokine production and neuroinflammation [20], [38], [39] (with some notable exceptions, see for instance [40]), in human macrophages and microglia, PGRN functions as a cytokine stimulator [35], [41]. Our previous study employing PGRN siRNA showed that in human microglia, PGRN plays a stimulator role for LPS-mediated TNFα and IP-10 production [35]. In no instances was PGRN found to be cytokine-suppressive in human microglia.

While these findings together point to the importance of maintaining PGRN levels for neuronal function and survival, the expression of PGRN has not been examined in HAND. Furthermore, no information is available on the expression or function of PGRN in the context of HIV disease even though PGRN expression is intricately linked to and regulated by inflammatory and infectious processes. The main *in vivo* objective of the current study was to measure PGRN in 107 matched CSF and plasma samples from a well-characterized cohort of people living with HIV disease and compare them to VL in plasma and CSF as well as to neurocognitive performance. The *in vitro* objective was to investigate the impact of HIV on PGRN production in microglia and the effects of PGRN on HIV replication. The results of the combined *in vivo* and *in vitro* experiments indeed provide strong evidence supporting a role for PGRN in the pathogenesis of HIV infection and HAND.

## Materials and Methods

### CHARTER Subjects

Matching CSF and plasma specimens were collected from 107 adults enrolled in the cross-sectional component of the CHARTER (CNS HIV AntiRetroviral Therapy Effects Research) cohort, a six-center, U.S. -based project funded by the National Institutes of Health. The project was approved by the institutional review board (IRB) of every participating institution (Johns Hopkins University, Mt. Sinai School of Medicine, University of California, San Diego, University of Texas Medical Branch-Galveston, University of Washington, Washington University). Informed written consent was obtained from all participants involved in the study.

The clinical and demographic data are shown in Table 1. The mean ± SD age was 43.7±8.7 years, 93 (86.9%) were male, 59 (55.1%) were white, 68 (63.6%) had AIDS diagnosis, 84 (78.5%) were taking ART, 58 (54.2%) had neurocognitive impairment. Fifty-three (49.5%) had undetectable plasma VL and 22 (20.8%) had current CD4+ T-cell counts <200/µL. Individuals with abnormal serum glucose, HCV seropositivity, or current substance use (based on urine drug screening) were excluded from the study. Of the subjects taking ART (n = 84), 53 (63.1%) had undetectable plasma VL and 74 (88.1%) had undetectable CSF VL. The median current CD4+ T-cell count was 396 (190–582). Of the subjects not taking ART (n = 23), median (interquartile range (IQR)) values for plasma and CSF VL were 18200 (1890–37600) and 1670 (103.3–5590), respectively. The median current CD4+ T-cell count was 493 (328–552) in the subgroup with no ART.

**Table 1:** Demographic and clinical characteristics.

*Characteristics*	*Subjects (N = 107)*
Age, years – Mean ± SD	43.7±8.7
Gender, Men – N (%)	93(86.9%)
Race, White – N (%)	59(55.1%)
AIDS – N (%)	68(63.6%)
ART use – N (%)	84(78.5%)
NC impairment – N (%)	58(54.2%)
Plasma HIV VL undetectable – N (%)	53(49.5%)
CSF HIV VL undetectable – N (%)	79(73.8%)
Current CD4+ T-cell count <200 cells/µL N (%)	22(20.8%)
Subjects taking ART	Subjects (n = 84)
Plasma HIV VL undetectable – N (%)	53(63.1%)
CSF HIV VL undetectable – N (%)	74(88.1%)
Current CD4+ T cell count (cells/µL) – median (IQR)^a^	396(190–582)
Subjects not taking ART	Subject (n = 23)
Plasma HIV VL (copies/mL) – median (IQR)^a^	18200(1890–37600)
CSF HIV VL (copies/mL) –median (IQR)^a^	1670(103.3–5590)
Current CD4+ T cell count (cells/µL) – median (IQR)^a^	493(328–552)

IQR^a^: interquartile range.

### Neurocognitive Assessment

All participants completed a comprehensive neurocognitive test battery that assessed seven cognitive domains commonly affected by HIV disease (speed of information processing, learning and memory, executive functions, language, working memory, and motor) [1], [42], [43]. The best available normative standards were used and corrected for the effects of age, education, gender and ethnicity. Test scores were converted to demographically corrected standard scores (T-scores) using available computer programs. To classify presence and severity of neurocognitive impairment, a published objective algorithm that has been shown to yield excellent interrater reliability in previous multisite studies [44] was applied. This algorithm conforms to the Frascati criteria for diagnosing HAND [3], which requires presence of at least mild impairment in at least two cognitive domains.

### PGRN and other Biomarker Quantifications

Concentrations of inflammatory mediators were determined in the plasma and CSF samples of all 107 subjects. A multiplex bead array quantified concentrations of IFNγ, IL-1β, IL-4, IL-6, IL-10, IL-13, IL-17, TNFα, CCL2/MCP-1, and CXCL10/IP-10 (EMD Millipore, Billerica, MA). PGRN ELISA was performed using human antibody DuoSet (DY2420, R&D Systems, Minneapolis, MN). All samples were diluted until the final concentrations were within the linear range of detection for the assay. All samples were tested in duplicates and concentrations interpolated from a standard curve constructed by 4-parameter fitting of internal standards. HIV RNA was measured by reverse transcriptase-polymerase chain reaction with a lower limit of quantitation (LLQ) of 50 copies/mL (Amplicor, Roche Diagnostics).

### Data Analysis for the Human Study

Inflammatory mediators whose levels were below the detection limit of the assay in >90% of samples were excluded from analysis. These were IL-1β, IL-4 and IL-13 for plasma and IL-1β, IL-4, IL-13, IFNγ and IL-17 for CSF. Undetectable HIV VL designates HIV RNA levels below 50 copies/ml, the lower limit of quantification (Amplicor). PGRN concentration values were log-transformed for plasma samples and square root transformed for CSF samples to normalize the data. Initial analysis of associations of age, gender, race, AIDS diagnosis, ART use, neurocognitive status, current CD4+ T cell count, and inflammatory mediators with plasma and CSF PGRN concentrations were examined using t-tests for categorical variables and Spearman correlation coefficients for continuous variables. Linear regression models or analysis of covariances (ANCOVA) were used to examine associations of PGRN with neurocognitive status adjusted for demographics, HIV VL, and inflammatory mediators. Variables with a p-value≤0.2 in initial analysis were included in multivariate analysis. Statistical analyses were performed using SAS (version 9.1, SAS Institute, Cary, NC) software.

### Primary Human Microglial Culture

Microglial cultures were prepared from human fetal abortuses (16–20 weeks gestational ages) as previously described [45] with minor modifications. All human tissue collection was approved by the Albert Einstein College of Medicine IRB (#: 1994-019). Informed written consent was obtained from all participants involved in the study. Primary mixed CNS cultures were prepared by enzymatic and mechanical dissociation of the cerebral tissue followed by filtration through nylon meshes of 230- and 130-µm pore sizes. Single cell suspension was plated at 1–10×10^6^ cells per ml in DMEM (Cellgro, now ThermoFisher Scientific) supplemented with 10% FBS (Gemini Bio-products, Woodland, CA), penicillin (100 U/ml), streptomycin (100 µg/ml) and amphotericin B (0.25 µg/ml) (complete medium) for 2 weeks, and then microglial cells were collected by aspiration of the culture medium. Monolayers of microglia were prepared in 60-mm tissue culture dishes at 1×10^6^ cells per 5 ml medium (for Q-PCR) or in 96-well tissue culture plates at 4×10^4^ per 0.1 ml medium (for ELISA). Four to eighteen hours later, cultures were washed to remove non-adherent cells (neurons and astrocytes). Microglial cultures were highly pure consisting of >98% Iba-1^+^ cells.

### HIV Infection

Microglial cultures were infected with single-cycle competent, vesicular stomatitis virus (VSVg) *env* pseudotyped HIV [46] or natural virus HIV_ADA_. VSVg *env* HIV was produced by cotransfecting 293T cells with pHIV_NL4.3_ (Nef-intact, Vpr-intact, Env-deficient, gift of Dr. Maurizio Federico [47]) and pVSVg *env*. Cells were infected with approximately 40 ng/ml p24 viral input that resulted in 25% to 50% cell infections at three days post inoculation [46], [48]. HIV_ADA_ was generated by transfecting 293T cells with pHIV_ADA_ obtained from Dr. Mario Stevenson, as previously described [49]. The HIV_ADA_ virus infects microglia through R5 *env*-CCR5 mediated fusion mechanism with HIV p24 production peaking at 14–21 days post inoculation [50], [51]. Input virus was washed out after 16 hours post-inoculation and then fresh medium was added back. Culture supernatants were collected with complete change of medium at 2 D, 4 D, 7 D and 14 D. In experiments with azidothymidine (AZT), a reverse transcriptase inhibitor, AZT was added back with each medium change.

### PGRN Knockdown by siRNA

Microglia were transfected with 20 nM control non-targeting small-interfering RNA (siRNA) or human PGRN-specific siRNA (Dharmacon, Chicago, IL) with transit-TKO transfection reagents from Mirus (Madison, WI) following the manufacturer's instructions and as previously described [35]. After incubation with siRNA for 2 to 4 days, cells were washed and then incubated with VSVg *env* HIV for 16 hours. Virus was then washed off the cells and fresh medium was added back. The cultures were further incubated for indicated time periods in complete medium. Knockdown efficiency was determined by PGRN ELISA or western blot.

### PGRN ELISA

The levels of PGRN in microglial culture supernatants were determined by ELISA as described for the human clinical specimens above and as previously described [35]. Microglial culture samples were diluted with fresh medium until the values fell within the linear range of the standard.

### Western blot

Western blot analysis was performed as previously described [35], [52] with minor modifications. Briefly, cultured cells in 60 mm dishes were scraped into lysis buffer (PBS plus protease inhibitors from Sigma). Thirty micrograms of protein was separated by 10% SDS-PAGE and then transferred to polyvinylidene difluoride membrane (Bio-Rad). The blots were blocked in PBS-0.1% Tween-20 containing 5% nonfat milk and then incubated with antibodies at 4°C for 16 h. Primary antibodies were rabbit anti-human PGRN (Invitrogen 40–3400 at 1∶100) or goat anti-human PGRN (R&D Systems AF2420 at 1∶1000) and goat anti-HIV p24 (Abcam, ab53841 at 1∶1000). β-actin (Sigma-Aldrich, A2228) was used as the loading control. The secondary antibodies were HRP-conjugated anti rabbit or anti-goat IgG (Pierce/Thermo Scientific, at 1∶1,000) applied for 1 h at RT. Signals were developed using West Pico or Femto chemiluminescent reagents (Pierce/Thermo Scientific). Densitometry was performed using the NIH ImageJ software.

### Data Analysis for Tissue Culture Studies

Differences in HIV infection-induced PGRN production were compared using ANOVA followed by Bonferroni post-hoc comparisons. Pooled data from siRNA experiments were analyzed by paired t-test. All statistics were performed using the GraphPad Prism 5.0 software.

## Results

### [1] Analyses of *Csf* Progranulin (Table 2–4)

#### Factors associated with CSF PGRN

Mean (± SD) PGRN levels were 129.7 (±8.3) ng/ml in plasma and 3.08 (±0.19) ng/ml in CSF, consistent with the reported ranges of PGRN levels in the body fluids [53]–[57]. We determined associations between PGRN concentrations and demographic and disease characteristics [42], [43]. The CSF data analyses are shown in Table 2. CSF PGRN levels (square-root transformed values) were significantly higher in the subgroup with detectable CSF VL (n = 28) than in the subgroup with undetectable VL (n = 79) (1.97±0.59 vs. 1.54±0.56, p<0.001). Gender, race, AIDS, ART use, neurocognitive status, and current CD4+ T-cell counts were not significantly associated with CSF PGRN (Table 2).

**Table 2:** Factors associated with CSF PGRN^a^.

*Factors*		*Mean*±*SD*	*P-value*
Gender	Male (n = 93)	1.67±0.61	ns
	Female (n = 14)	1.49±0.54	
Race	Black (n = 34)	1.50±0.76	ns
	Other (n = 14)	1.72±0.55	
	White (n = 59)	1.72±0.50	
AIDS	No (n = 39)	1.76±0.71	ns
	Yes (n = 68)	1.59±0.52	
ART Use	No (n = 23)	1.77±0.81	ns
	Yes (n = 84)	1.62±0.53	
Neurocognitive status	Impaired (n = 58)	1.59±0.64	ns
	Normal (n = 49)	1.72±0.54	
CSF HIV VL	Non-detectable (n = 79)	1.54±0.56	<0.001
	Detectable (n = 28)	1.97±0.59	
Current CD4+ T-cell count	<200 (n = 22)	1.64±0.61	ns
	> = 200 (n = 84)	1.65±0.60	

a CSF PGRN was square-root transformed,

ns: not significant.

#### Correlation analyses of CSF PGRN and other inflammatory mediators and VL

We next examined whether the levels of PGRN correlated with levels of other inflammatory mediators in the CSF (n = 107). CSF PGRN levels positively correlated with IL-10 (r = 0.25, p = 0.009), IP-10 (r = 0.37, p<0.001) and TNFα (r = 0.42, p<0.001), and inversely correlated with current CD4+ T-cell counts (r = −0.21, p = 0.034). No correlations were found with IL-6, MCP-1 or age (Table 3, left column).

**Table 3:** Correlation of CSF PGRN and other factors.

	All subjects (n = 107)		Subjects with Undetectable CSF VL (n = 79)		Subjects with detectable CSF VL (n = 28)	
	*r*	*p-value*	*r*	*p-value*	*r*	*p-value*
Age	0.15	ns	0.12	ns	0.48	0.05
Current CD4+ count	−0.21	0.034	−0.13	ns	−0.10	ns
CSF HIV	N/A	N/A	N/A	N/A	0.33	ns
IL-6	0.08	ns	0.05	ns	0.13	ns
IL-10	0.25	0.009	0.09	ns	0.48	0.01
IP-10	0.37	<0.001	0.12	ns	0.69	<0.001
MCP-1	0.18	ns	−0.06	ns	−0.28	ns
TNFα	0.42	<0.001	0.26	0.05	0.46	<0.001

*r*: Spearman correlation coefficient, ns: not significant.

N/A: analysis is not applicable due to measurable VL in only 28 subjects.

Stratifying the analysis by CSF VLs identified that these correlations were stronger in those with detectable VLs. In the HIV detectable group (n = 28), a stronger correlation between CSF PGRN and IP-10 (r = 0.69, p<0.001), IL-10 (r = 0.48, p = 0.01) and TNFα (r = 0.46, p<0.001) was found than in the HIV undetectable group (n = 79) (Table 3, middle and right columns). There was also a positive correlation between age and CSF PGRN in subjects with detectable CSF VL (p = 0.05). There was no significant correlation between CSF VL and CSF PGRN levels in the 28 subjects with detectable CSF VL. In the HIV undetectable group, a significant correlation was found only with TNFα (r = 0.26, p = 0.05).

#### Multivariate analyses

Multivariate linear regression analysis of factors associated with CSF PGRN is shown in Table 4. There was a trend toward lower CSF PGRN in individuals with neurocognitive impairment (β = −0.174, p = 0.095) after adjusting for CSF VL, age and TNFα in all subjects (left column). Higher CSF PGRN was associated with older age (β = 0.013, p = 0.034), detectable CSF VL (β = 0.281, p = 0.033) and higher TNFα (β = 0.076, p<0.001) (left column).

**Table 4:** Multivariate linear regression analysis of factors associated with CSF PGRN.

*Characteristics*	*All subjects (n = 107)*		*Subjects with undetectable CSF VL (n = 79)*		*Subjects with detectable CSF VL (n = 28)*	
	aβ-coefficient (SE)	p-value	aβ-coefficient (SE)	p-value	aβ-coefficient (SE)	p-value
Neurocognitive impairment	−0.174 (0.103)	0.095	−0.253 (0.124)	0.046	0.048 (0.192)	0.805
Age, per year	0.013 (0.006)	0.034	0.013 (0.008)	0.094	0.015 (0.012)	0.203
Detectable CSF VL	0.281 (0.130)	0.033	N/A	N/A	N/A	N/A
TNFα	0.076 (0.021)	<0.001	0.075 (0.028)	0.009	0.080 (0.032)	0.020

a β-coefficient less than zero indicate an inverse association between the characteristic and PGRN.

N/A: not applicable.

In the subgroup of 79 subjects with undetectable CSF VL, neurocognitive impairment was associated with lower CSF PGRN levels (β = −0.253, p = 0.046) (Table 4, middle column). Higher TNFα was associated with higher CSF PGRN (β = 0.075, p = 0.009). Age was not associated with CSF PGRN (β = 0.013, p = 0.094). In 28 subjects with detectable CSF VL, higher TNFα was associated with higher CSF PGRN (β = 0.080, p = 0.020) (Table 4, right column). Neurocognitive impairment and age were not associated with CSF PGRN in this group.

### [2] Analyses of *Plasma* Progranulin (Table 5–7)

Plasma PGRN levels (log transformed values) were significantly higher in subjects with detectable plasma VLs (n = 54) than in those with undetectable plasma VLs (n = 53) (4.84±0.67 vs. 4.50±0.53, p = 0.003) (Table 5). Higher plasma PGRN levels were also associated with current CD4+ T-cell counts below 200/µL (5.04±0.49 vs. 4.58±0.63, p = 0.002). Gender, race, AIDS status, ART use, and neurocognitive status were not associated with plasma PGRN (Table 5).

**Table 5:** Factors associated with plasma PGRN^a^.

*Factors*		*Mean* ± *SD*	*P-value*
Gender	Male (n = 93)	4.68±0.65	ns
	Female (n = 14)	4.63±0.40	
Race	Black (n = 34)	4.61±0.66	ns
	Other (n = 14)	4.82±0.47	
	White (n = 59)	4.68±0.64	
AIDS	No (n = 39)	4.63±0.74	ns
	Yes (n = 68)	4.70±0.55	
ART Use	No (n = 23)	4.81±0.77	ns
	Yes (n = 84)	4.63±0.58	
Neurocognitive status	Impaired (n = 58)	4.67±0.64	ns
	Normal (n = 49)	4.67±0.62	
CSF HIV VL	Non-detectable (n = 53)	4.50±0.53	0.003
	Detectable (n = 54)	4.84±0.67	
Current CD4+ T-cell count	<200 (n = 22)	5.04±0.49	0.002
	> = 200 (n = 84)	4.58±0.63	

a Plasma PGRN was log transformed,

ns: not significant.

#### Correlates of plasma PGRN

Higher plasma PGRN levels correlated with lower current CD4+ T-cell counts (r = −0.50, p<0.001) and higher IP-10 (r = 0.49, p<0.001) (Table 6, left column). There was no correlation between plasma PGRN and age, plasma IFNγ, IL-6, IL-10, IL-17, MCP-1 or TNFα. The correlations with either IP-10 (r = 0.59, p<0.001) or CD4+ T-cell counts (r = −0.58, p<0.001) strengthened in the detectable HIV subgroup (n = 54) (right column). There was also a correlation between plasma VL and plasma PGRN levels in subjects with detectable VL (r = 0.47, p<0.001) (right column). In subjects with undetectable plasma VL (n = 53) (middle column), the correlations with IP-10 and CD4+ T-cell counts still existed, though much weaker than in subjects with detectable plasma VL.

**Table 6:** Correlation of plasma PGRN and other factors.

	*All subjects (n = 107)*		*Subjects with undetectable plasma VL (n = 53)*		*Subjects with detectable plasma VL (n = 54)*	
	*r*	*p-value*	*r*	*p-value*	*r*	*p-value*
Age	0.00	ns	0.00	ns	0.09	ns
Current CD4+ count	−0.50	<0.001	−0.38	0.005	−0.58	<0.001
Plasma HIV VL	N/A	N/A	N/A	N/A	0.47	<0.001
IFN-γ	0.05	ns	0.02	ns	−0.02	ns
IL-6	0.14	ns	0.01	ns	0.26	ns
IL-10	0.17	ns	0.03	ns	0.23	ns
IL-17	0.05	ns	0.00	ns	0.06	ns
IP-10	0.49	<0.001	0.29	0.037	0.59	<0.001
MCP-1	0.16	ns	0.22	ns	0.10	ns
TNFα	0.15	ns	−0.05	ns	0.22	ns

*r:* Spearman correlation coefficient, ns: not significant.

N/A: not applicable.

#### Multivariate analyses

Multivariate modeling identified that higher plasma PGRN levels were associated with lower current CD4+ T-cell count (β = −0.433, p = 0.001), and detectable plasma VL (β = 0.231, p = 0.031) (Table 7, left column). Neurocognitive impairment (β = −0.021, p = 0.838) was not associated with plasma PGRN. A significant interaction between detectable plasma VL and IP-10 was present, indicating that the relationship between plasma IP-10 and plasma PGRN were modified by detectable plasma VL. To adjust for this effect modification, subjects with detectable plasma VL and undetectable plasma VL were analyzed separately (Table 7, middle and right columns).

**Table 7:** Multivariate linear regression analysis of factors associated with plasma PGRN.

*Characteristics*	*All subjects (n = 107)*		*Subjects with undetectable plasma VL (n = 53)*		*Subjects with detectable plasma VL (n = 54)*	
	aβ-coefficient (SE)	p-value	aβ-coefficient (SE)	p-value	aβ-coefficient (SE)	p-value
Neurocognitive impairment	−0.012 (0.102)	0.838	−0.094 (0.139)	0.504	−0.132 (0.146)	0.368
Current CD4+ count >200	−0.433 (0.126)	0.001	−0.436 (0.172)	0.001	−0.439 (0.185)	0.021
Detectable plasma VL	0.231 (0.105)	0.031	N/A	N/A	N/A	N/A
IP-10^b^	0.105 (0.096)	0.280	0.104 (0.094)	0.278	0.363 (0.065)	<0.001
Detectable plasma VL*IP-10^c^	0.250 (0.115)	0.032	N/A	N/A	N/A	N/A

a β-coefficients less than zero indicate an inverse association between the characteristic and PGRN.

b IP-10 was subtracted from mean = 1603.4 and divided by SD = 1371.4 to reduce collinearity between IP-10 and detectable plasma VL.

c Interaction between IP-10 and detectable plasma VL.

N/A: not applicable.

In 53 subjects with undetectable plasma VL, current CD4+ T-cell count >200 was associated with lower plasma PGRN (β = −0.436, p = 0.001). Neurocognitive impairment and IP-10 were not associated with plasma PGRN in this group. In 54 subjects with detectable plasma VL, current CD4+ T-cell count >200 was associated with lower plasma PGRN (β = −0.439, p = 0.021). Higher IP-10 was associated with higher plasma PGRN only in this subgroup (β = 0.363, p<0.0001). Neurocognitive impairment was also not associated with plasma PGRN in this group. *These data suggest that, in plasma, IP-10, PGRN, and VL are closely related.*

### [3] *In vitro* Studies of HIV-induced PGRN Production and Function in Human Microglia (Figures 1 and 2)

#### HIV infection induces PGRN in human microglia

**Figure 1:**
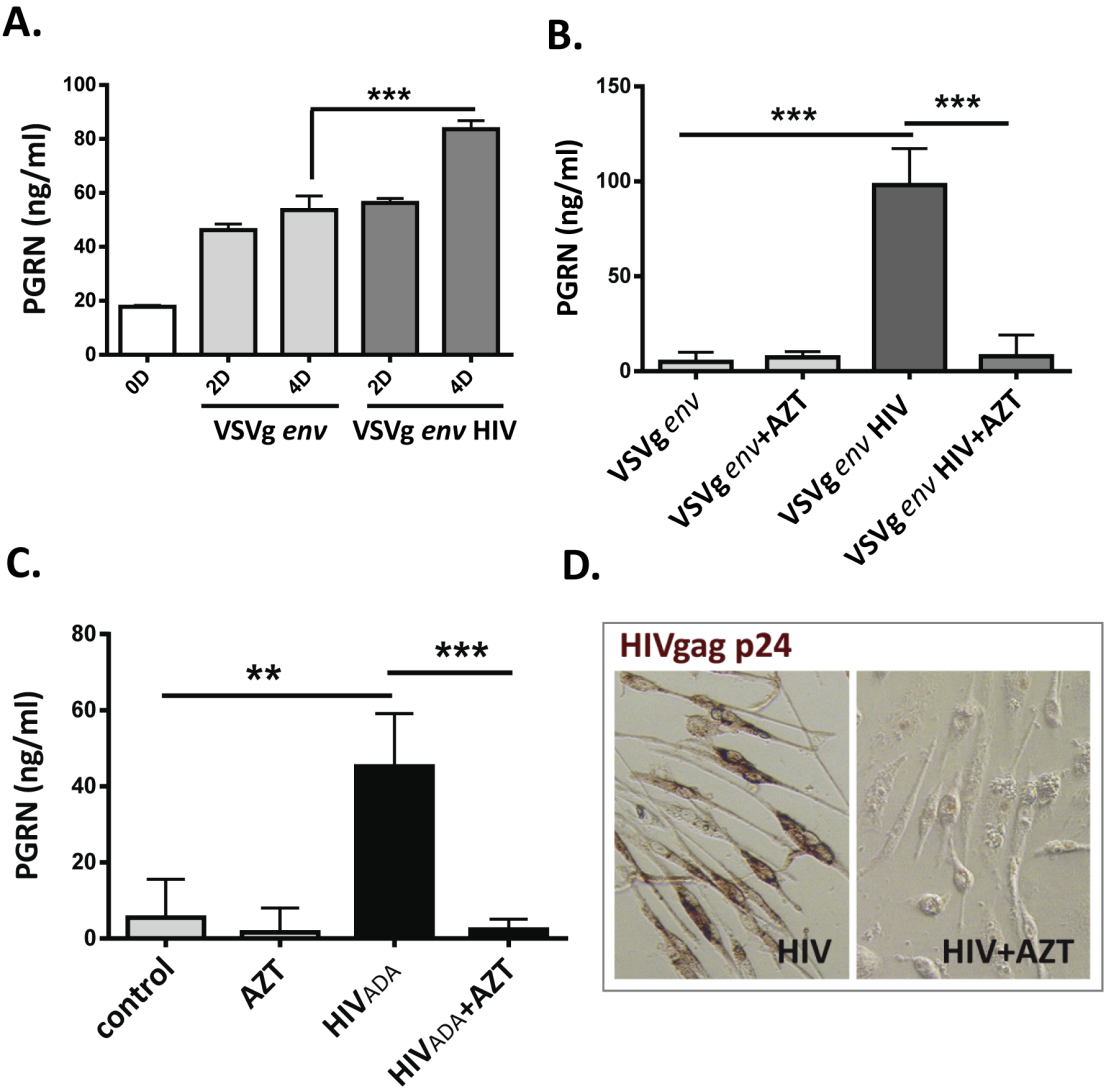
HIV infection induces PGRN production in microglia. Primary human microglial cells were inoculated with VSVg *env* HIV or HIV_ADA_ and PGRN in culture supernatants were determined by ELISA as described in the Materials and Methods. Control cultures were treated with VSVg *env* protein or mock infected. (A) Results with VSVg *env* HIV are shown. Culture supernatants were collected at 2 D and 4 D with complete change of medium (mean ± SD from triplicate cultures) (B) Microglia were incubated with AZT (10 µg/ml) or vehicle for 1 h, then exposed to VSVg *env* HIV as in A. PGRN was measured at 7 D and 14 D with complete change of medium. Data shown are accumulation between 7D–14D (mean ± SD, n = 3). (C) HIV_ADA_ (HIV *env* bearing virus) was used to determine PGRN production as described in the Materials and Methods. Data shown are PGRN accumulation in culture supernatants between 7D–14D (mean ± SD, n = 3) (D) Microglial cultures infected with VSVg *env* HIV in B showing cell viability, *gag* p24 expression, and complete suppression of p24 by AZT treatment. **P<0.01, ***P<0.001 by ANOVA followed by Bonferroni correction.

Based on clinical data that indicate strong association between PGRN expression and HIV infection [30] (and current study), we directly examined whether HIV modulates PGRN expression in our well-characterized human microglial culture system [50], [52]. Microglia were infected with HIV_ADA_ or VSVg *env*-pseudotyped HIV and PGRN levels were determined by ELISA and western blot analysis as previously described [47], [58]. The effect of azidothymidine (AZT), a reverse transcriptase inhibitor, was examined to determine whether active viral replication was required for PGRN modulation. These experiments showed that exposure to either type of HIV (HIV *env*- or VSVg *env*-bearing) induced PGRN in a time-dependent manner and that AZT abolished HIV-mediated PGRN induction in both cultures (Figure 1). *These results show that PGRN is induced in microglia by HIV infection and that this requires productive infection.*

#### PGRN inhibits HIV replication in microglia

**Figure 2:**
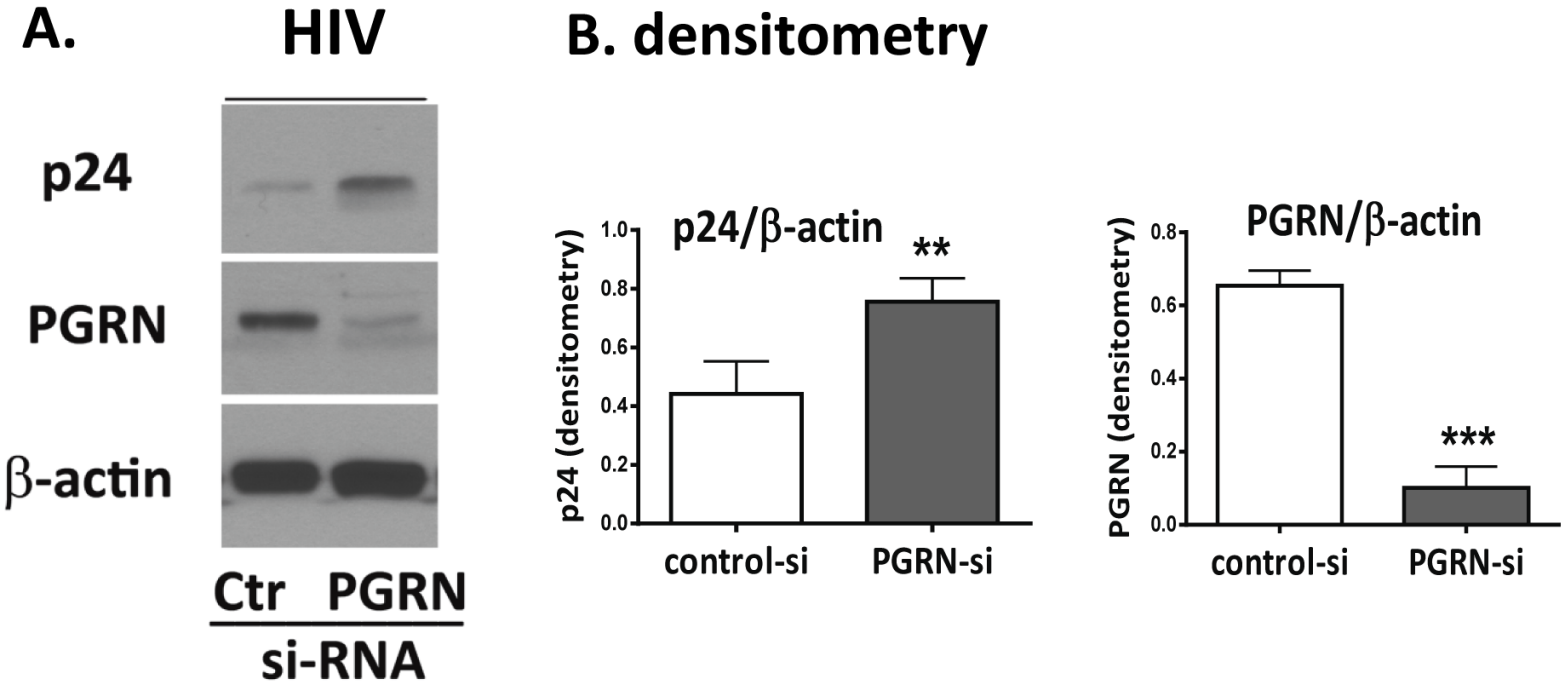
PGRN is an endogenous anti-HIV factor. Microglial PGRN was knocked down using RNAi 2–4 days prior to VSVg *env* HIV exposure as described in the Materials and Methods. Control cultures were treated with control, irrelevant siRNA (Ctr). The amounts of HIV (p24) and PGRN expression were determined by western blot analyses. (A) A representative western blot showing suppression of PGRN and increase of p24 following PGRN siRNA treatment. (B) Pooled densitometry data from four independent experiments showing significant inhibition of PGRN and increase of HIV (*gag* p24 express) in microglial cells treated with PGRN siRNA (vs. control siRNA). **P<0.01, ***P<0.001 by paired t-test.

PGRN is mainly a product of innate immune cells and, while its antimicrobial capacity has been suggested, this has not been directly examined. We therefore examined whether microglial PGRN affects the level of HIV replication. Microglial cells were first transfected with PGRN siRNA or control (Ctr) siRNA for 2–4 days and then inoculated with VSVg *env* HIV for an additional 4 days. Western blot analysis was performed to determine the amount of HIV*gag* (p24) and PGRN expression in each culture. Representative western blot and pooled densitometry data are shown in Figure 2A and B, respectively. siRNA-induced PGRN knockdown was highly efficient in microglia. Furthermore, in PGRN siRNA-treated cultures, HIVgag expression was significantly increased, indicating that PGRN has an HIV-suppressive role in these cultures. *These results demonstrate that PGRN functions as an endogenous inhibitor of HIV replication in human microglia. Together, these results show, for the first time, that PGRN is an innate anti-HIV protein.*

## Discussion

Despite emerging evidence for the role of PGRN in antimicrobial immune function, studies that directly examined this role in infectious diseases are rare [20], [37], [59]. The observation that PGRN is primarily a product of cells of the innate immune system (neutrophils, monocytes and macrophages) further suggests this role [20], [35], [40]. Previous studies have found that CNS clearance of *Listeria monocytogenes* is delayed in PGRN knockout mice [20]. PGRN expression was induced in gastric epithelial cells infected with *Helicobacter pylori* in a manner requiring direct contact with live bacteria. *H. pylori*-infected subjects also have increased serum PGRN levels [60], [61]. These results together suggest that increased PGRN expression is a natural response of gastric epithelial cells to bacterial infection.

To our knowledge, ours is the first study that demonstrated the role of PGRN in viral infection. PGRN was induced by HIV infection of microglia in a manner dependent on active viral replication. Furthermore, siRNA studies revealed that PGRN functions as an innate anti-HIV factor in infected cells. These *in vitro* findings are supported by the human biomarker study which showed a strong correlation between plasma PGRN levels and plasma VL. Together, these results provide first direct evidence that PGRN is an innate antiviral molecule produced by infected host cells, assigning a new role for this multifunctional factor. Our results also provide a biological basis for our previous observation that PGRN (mRNA and protein) are increased in HIVE [30].

Another significant finding in this study is the association between reduced CSF PGRN levels and neurocognitive impairment in subjects with undetectable VL. These results suggest that PGRN has dual mechanisms of regulation in HIV-infected individuals. With viral replication, PGRN production is actively induced in infected cells and acts as an innate antiviral molecule. In individuals with viral suppression, the stimulus for PGRN production no longer exists. In these individuals, various environmental and genetic factors can lead to reduced PGRN production [21], [24], [30], [35] (also see below). The loss of PGRN’s neuroprotective properties could then increase vulnerability to neuronal injury and neurocognitive impairment.

One of the clearly defined mechanisms for growth factor depletion is the proinflammatory environment. For example, the production of several neurotrophic factors (PGRN, IGF-1 and BDNF) is reciprocally regulated by the Th1/M1 and Th2/M2 cytokines [62]–[65]. Specifically, strong proinflammatory activators such as LPS suppress growth factor production in macrophages and microglia. Reduction in CSF PGRN may thus reflect chronic immune activation and neuroinflammation that has been recognized as a risk factor for neurocognitive complications during suppressive ART. The degree of CSF PGRN depletion (∼23% on average) in cognitively impaired (vs. unimpaired) individuals within the HIV-undetectable subgroup is likely biologically significant, given that normal CSF PGRN concentrations are much lower than those of plasma (∼1/40) and that normal aging and cognition is critically dependent on the normal concentrations of PGRN, as shown by fatal neurodegeneration in humans caused by genetic 50% deficiency. We propose that longitudinal determinations of CSF PGRN may be useful as a prognostic marker of HAND. If our findings are confirmed, then investigations of PGRN replacement therapy for HAND may have merit.

Our study also shows *in vitro-in vivo* concordance between PGRN and TNFα and IP-10 reflecting our published data from cultured microglia [35]. *In vitro*, TNFα was the most persistently and profoundly affected cytokine by PGRN knockdown among many tested, suggesting their co-regulation following LPS stimulation. IP-10 was the second most significantly co-regulated cytokine. Although the inducing signal(s) for these cytokines *in vivo* are unknown, our human data strengthen the notion that PGRN is a molecule closely related to TNFα and IP-10 as well as IL-10 (especially in the CSF). For example, of the nine soluble proteins measured, our analyses showed significant correlations between CSF PGRN and TNFα, IP-10 and IL-10. CSF VL also showed a positive correlation with TNFα and IL-10 with trends for IP-10 and PGRN. Analysis of the plasma showed similar findings. Correlation between plasma PGRN and IP-10 was strongest in subjects with detectable plasma VLs, indicating the effect of active HIV replication.

**Figure 3:**
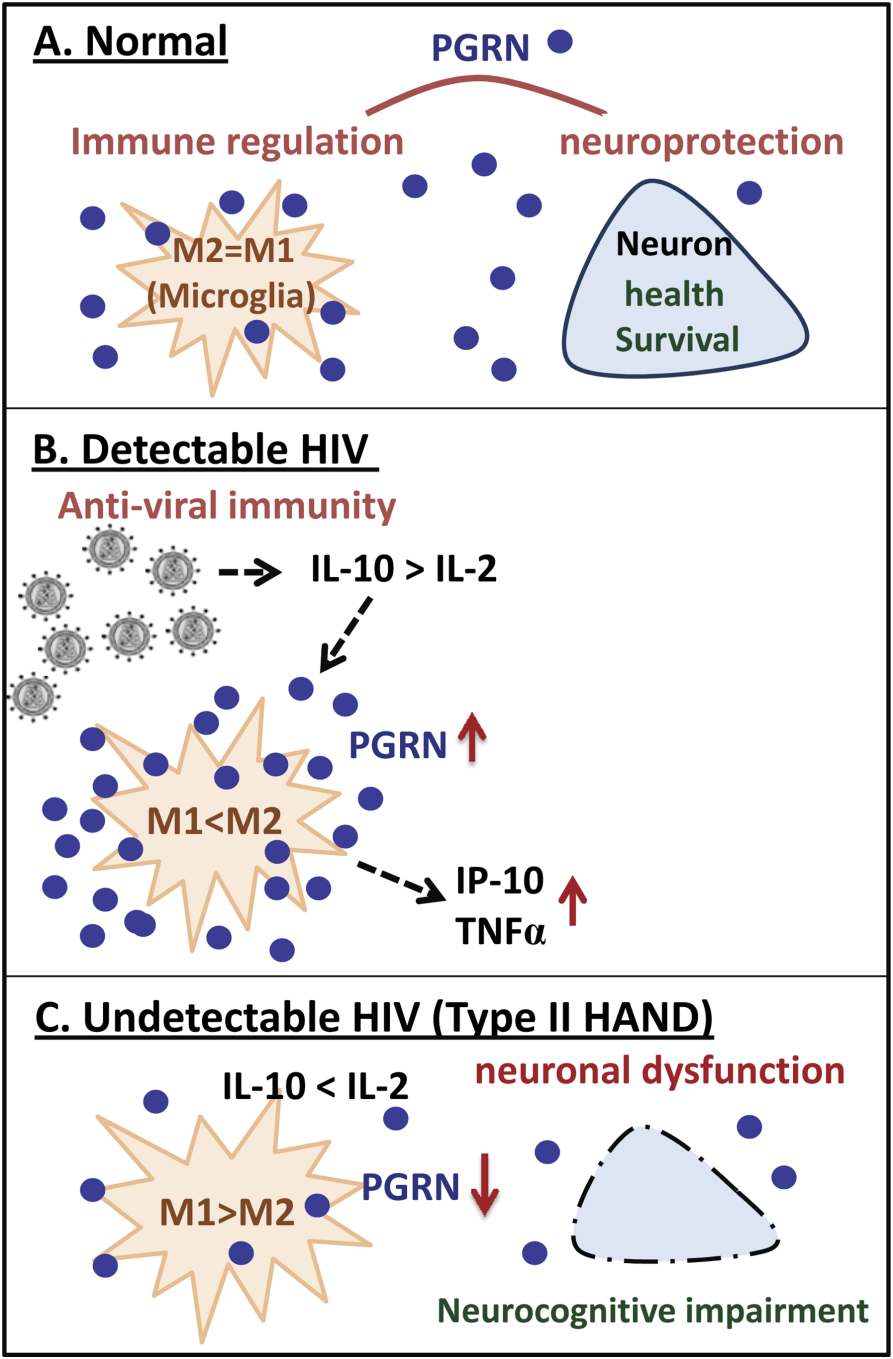
Hypothetical scenario linking HIV, inflammation, PGRN and neurocognition based on our data and literature. (A) under normal homeostatic conditions, PGRN is produced by neurons and microglia and contributes to neuroprotection and immune balance. (B) In HIV+ individuals with detectable HIV replication, viral infection contributes to M2 predominant macrophage/microglial cytokine environment (M2>M1) in part due to IL-10 production and CD4+ T cell loss. This environment favors PGRN overproduction. IP-10 and TNFα are also co-regulated with PGRN, possibly downstream of PGRN, though the mechanisms are unclear. PGRN contributes to innate antiviral immunity in HIV-infected macrophages. (C) In HIV+ individuals with undetectable virus, macrophages are under M1 predominant (M1>M2) conditions which decrease the production of PGRN. Reduction of PGRN in the CSF/CNS compartment in which the amount of PGRN is limiting, neuronal function and survival is further compromised contributing to neurocognitive impairment (“Type II HAND”) (See text for detail).

Given the opposing roles the M1 and M2 mediators play in macrophage PGRN production, we initially asked whether PGRN levels correlate with those of M1 and M2 cytokines in plasma or CSF. While our data do not clearly support the role of M1 and M2 cytokines in PGRN production, the association with IL-10 implicates the Th2/M2 response. Previous studies identified that IL-10 is upregulated in multiple cell types during HIV infection [66] and that Th2 rather than Th1 cytokine profiles were generally associated with progressive HIV infection [67]–[69]. Our conclusions about Th2 and Th1 profiles are limited by the absence from our panel of IL-2, the prototype Th1 cytokine produced by T cells. However, the association between PGRN and CD4+ T-cell counts indirectly supports a link with IL-2 (fewer CD4+ T-cells, less IL-2 and more PGRN). We speculate that PGRN production and the dominant effect of PGRN differs based on whether HIV is replicating or suppressed. This may reflect in part the cytokine environment associated with productive and non-productive HIV infection, with the Th2/M2 dominant environment promoting PGRN and the Th1/M1 dominant environment suppressing PGRN production. Our hypothesis based on available data and literature is shown in Figure 3.

Further studies are needed to determine whether longitudinal CSF PGRN determinations are useful as a prognostic marker for HAND in the CSF VL undetectable subgroup. Whether common PGRN genetic variations associated with significant PGRN deficiency [24]–[26] predispose individuals to HAND should also be determined. Furthermore, given multiple neuronal growth factors are produced by microglia and macrophages in the CNS with similar immunologic regulatory mechanisms, future studies should determine whether multiple growth factor deficiencies underlie neurocognitive impairment in HAND. Additional studies support the idea of growth factor deficiency in HAND, as well [13], [70]. Immune therapies that promote growth factor production or direct growth factor replacement therapies can also be considered.
